# A paraventricular thalamus to insular cortex glutamatergic projection gates “emotional” stress-induced binge eating in females

**DOI:** 10.1038/s41386-023-01665-6

**Published:** 2023-07-20

**Authors:** Roberta G. Anversa, Erin J. Campbell, Leigh C. Walker, Sarah S. Ch’ng, Muthmainah Muthmainah, Frederico S. Kremer, Amanda M. Guimarães, Mia J. O’Shea, Suheng He, Christopher V. Dayas, Zane B. Andrews, Andrew J. Lawrence, Robyn M. Brown

**Affiliations:** 1 Department of Biochemistry and Pharmacology, University of Melbourne, Parkville, Australia; 2 The Florey Institute of Neuroscience and Mental Health, Mental Health Division, Parkville, Melbourne Australia; 3 The Florey Department of Neuroscience and Mental Health, University of Melbourne, Parkville, Melbourne Australia; 4 School of Biochemical Sciences and Pharmacy, University of Newcastle, Newcastle, Australia; 5 Department of Anatomy, Faculty of Medicine, Universitas Sebelas Maret, Surakarta, Indonesia; 6 Laboratório de Bioinformática, Programa de Pós-Graduação em Biotecnologia, Centro de Desenvolvimento Tecnológico, Federal University of Pelotas, Pelotas, Brazil; 7 Biomedicine Discovery Institute and department of Physiology, Monash University, Clayton, Australia

**Keywords:** Feeding behaviour, Motivation, Insula

## Abstract

It is well-established that stress and negative affect trigger eating disorder symptoms and that the brains of men and women respond to stress in different ways. Indeed, women suffer disproportionately from emotional or stress-related eating, as well as associated eating disorders such as binge eating disorder. Nevertheless, our understanding of the precise neural circuits driving this maladaptive eating behavior, particularly in women, remains limited. We recently established a clinically relevant model of ‘emotional’ stress-induced binge eating whereby only female mice display binge eating in response to an acute “emotional” stressor. Here, we combined neuroanatomic, transgenic, immunohistochemical and pathway-specific chemogenetic approaches to investigate whole brain functional architecture associated with stress-induced binge eating in females, focusing on the role of Vglut2 projections from the paraventricular thalamus (PVT^Vglut2+^) to the medial insular cortex in this behavior. Whole brain activation mapping and hierarchical clustering of Euclidean distances revealed distinct patterns of coactivation unique to stress-induced binge eating. At a pathway-specific level, PVT^Vglut2+^ cells projecting to the medial insular cortex were specifically activated in response to stress-induced binge eating. Subsequent chemogenetic inhibition of this pathway suppressed stress-induced binge eating. We have identified a distinct PVT^Vglut2+^ to insular cortex projection as a key driver of “emotional” stress-induced binge eating in female mice, highlighting a novel circuit underpinning this sex-specific behavior.

## Introduction

Eating disorders are highly deleterious mental health conditions with one of the highest mortality rates worldwide [1]. Eating disorders are profoundly influenced by sex, with reported estimates of the male-to-female ratio of eating disorder prevalence ranging from 1:4 up to 1:9 [2]. Moreover, subclinical disordered eating behaviors [3], as well as anxiety and depressive symptoms are also more prevalent in females [4, 5]. Accordingly, negative emotions such as stress, anger, and low mood are more likely to drive overeating in women [6]. Poor understanding of the precise biological mechanisms underlying this sex-specific behavior likely underpins the high failure rate of treatments, both for overweight and obesity driven by overconsumption, as well as for binge eating disorder (BED) [2, 7].

Although psychosocial factors such as increased appearance and weight pressures in women are established contributors to sex-specific risk for eating disorders, valuable work with animal models has begun to establish a biological basis for this sex difference [8]. We recently developed a mouse model whereby exposure to an acute ‘emotional’ stressor induced binge eating specifically in female mice without the need for caloric restriction [9]. The ability of this model to recapitulate the sex difference observed in humans provides us with the unique ability to probe the neural correlates of this behavior [10].

The insula is a brain region ideally positioned to govern emotional stress-driven eating given its established role in appetite, motivated behavior and emotional processing [11–14]. Greater activation of this region in response to photographs of high- vs. low- calorie foods is observed specifically during negative affective states in women [15], and also in normal weight women as compared to men [16]. In clinical populations, women with high BMI, including those with YFAS-defined food addiction, consistently show greater insular activation in response to food cues compared to normal weight women [17–20]. Further, this activation has been reported to predict craving prior to binge eating in women with bulimia nervosa [21].

Cross-sectional human imaging studies do not, however, address whether heightened insular activation is pre-existing in nature or a consequence of overconsuming palatable food. Rodent studies have provided some insight in this regard; for example, intermittent access to a palatable diet induces functional adaptations in insular pyramidal neurons [22] and targeted optogenetic inhibition of glutamatergic efferents of the anterior insula to nucleus accumbens (NAc) reduces compulsive food self-administration in female rats [23]. Recent evidence indicates that the insula receives dense glutamatergic innervation from various brain loci involved in reward and appetitive motivation [24], suggesting that glutamatergic projections to the insula constitute prime candidates that drive stress-induced binge eating. Nevertheless, glutamatergic afferents to the insula are yet to be evaluated in models of disordered overeating.

Here, we examined the role of inputs to the insular cortex in *Vglut2*-Cre female mice in our recently developed model of stress-induced binge eating [9]. We first combined retrograde viral tracing with Fos-protein immunohistochemistry in *Vglut2-Cre* mice to examine neuronal activation associated with stress-induced binge eating in insular cortical afferents. Stress-induced binge eating was associated with selective activation of a Vglut2 projection from the paraventricular thalamus (PVT^Vglut2+^) to insular cortex. We mapped whole-brain Fos expression and performed hierarchical clustering of Euclidean distances, which revealed a shift in the organizational patterns of Fos co-activation in stress-binge mice, indicating a disrupted relationship between brain regions, particularly those associated with stress and reward pathways. To test causal involvement of glutamatergic projections from the PVT to insular cortex we used pathway-specific chemogenetics to selectively inhibit PVT^Vglut2+^-insula projections during stress-induced binge eating. Selective inhibition of this pathway reduced stress-induced overconsumption of palatable food in female mice without affecting locomotor activity or anxiety-like behavior. Collectively, our findings highlight a novel circuit that drives stress-induced bingeing in females.

## Materials and Methods

See Supplementary Information for detailed materials and methods.

### Animals and housing

*Vglut2*-Cre (Slc17a6^tm2(cre)Lowl^/J [25]) mice were 7–8 weeks old at the start of experiments; total *n* = 71). *Vglut2*-Cre parental stock was obtained from Zane Andrews (Monash University), originally purchased from The Jackson Laboratory (stock #016963), see Fig. S1 for validation of Cre expression. All mice were single-housed with rooms maintained on a reverse 12 h light/dark cycle (lights off 0700). Room temperature maintained at 21–22.5 °C, humidity at 45–60%. All experiments performed 2 h after dark-cycle commencement. Mice received *ad libitum* access to standard chow (4.5% fat; Barastoc, Ridley Corporation, Australia) and water unless otherwise specified. All procedures were conducted in adherence to the Prevention of Cruelty to Animals Act (2004), under the guidelines of the NHMRC Code of Practice for the Care and Use of Animals for Experimental Purposes in Australia (2013) and were approved by Florey Ethics Committee.

### Stress-induced binge eating protocol

**Fig. 1:**
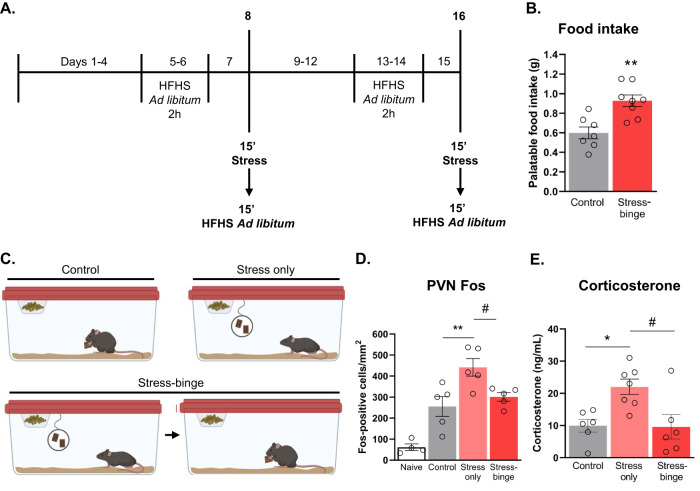
Palatable food intake following stress-binge paradigm and validation of stressor. **A** Behavioral timeline. **B** Stressed mice consumed significantly more palatable food in the 15 min following stress compared to control mice who were not subjected to the stressor but had free access to palatable food for the same period. ^**^p = 0.0019, unpaired t-test, two-tailed, *df* = 13. Data from day 16. **C** Schematic of distinct behavioral manipulations for control, stress only, and stress-binge groups. **D** Fos expression following behavioral manipulation across different groups. ^**^p = 0.0061, ^#^*p* = 0.0420. **E** Serum corticosterone levels following stress-binge protocol. *p = 0.0178, ^#^*p* = 0.0148. One-way ANOVA with Tukey post hoc multiple comparisons. Naïve *n* = 3, control *n* = 6-7, stress only *n* = 5, stress-binge *n* = 7–8. Data are presented as mean ± SEM. 15’, 15 min; 2 h, 2 h; HFHS, high-fat high-sugar.

Detailed protocol in supplemental methods. Briefly, mice are provided intermittent access to a highly palatable food reward in their homecage before being subjected to an ‘emotional’ stressor: an enclosed tea strainer containing palatable food [9]. Mice have visual and olfactory access to the food but could not consume it for 15 min. Following this, mice from the stress-binge group are allowed to freely consume the food for 15 min. Control mice have access to the food reward for 15 min but no stressor (Fig. 1).

### Serum collection and corticosterone quantification

A separate cohort of female mice (*n* = 21) were subjected to the behavioral protocol described above for corticosterone quantification. On day 16 of the behavioral protocol, mice were euthanized via cervical dislocation immediately after behavioral manipulation and blood collected for corticosterone quantification. Refer to supplemental materials for detailed methods.

### Stereotaxic surgeries

*Retrograde tracer surgeries*: mice (*n* = 28) received unilateral injections of 140 nL Cre-dependent retrograde virus AAV2-EF1a-DO_DIO_TdTomato_EGFP-WPRE-pA titer ~7 × 10^12^/100 μL; Addgene Massachusetts, USA, plasmid #37120). Injections targeted medial insular cortex: AP: +0.86 mm, ML: +/−3.55-3.60 mm, DV: −4.0 mm.

*Chemogenetic surgeries:* either Cre-dependent inhibitory designer receptors exclusively activated by designer drugs (DREADD) AAV2-hSyn-DIO-hM4D(Gi)-mCherry (110 nL; titer ~5 × 10^12^/100 μL; Addgene) or control virus AAV1/2-CAG-FLEX-tdTomato (tdT, 110 nL; ~8.7 × 10^11^ vg/ml; prepared by S. Layfield and R. Bathgate, Florey) were stereotaxically injected bilaterally into PVT (*n* = 40) at 100 nL/min rate (Nanoject III) plus 5 min. PVT co-ordinates: AP −1.59 mm, ML + /−1.0 mm, DV −2.96 mm, 20° angle (adapted from [26]). Bilateral guide cannulae (26 gauge, 3.9 mm BioScientific, Australia) were also implanted above insula: AP + 0.86 mm, ML + /−3.55 to 3.60 mm, DV −3.90 mm) and anchored to the skull with jeweler’s screws and dental cement.

### Fos-protein expression in insular cortex afferents after stress-induced binge eating

After recovery, mice were randomly assigned to 4 different groups (control (*n* = 8), stress only (*n* = 8), stress-binge (*n* = 8), and naïve (*n* = 4)) and were subjected to the stress-binge protocol (9). Amount consumed by stress-binge versus control animals was also compared. 90 min after testing, mice were perfused and brains processed for immunohistochemistry.

### Chemogenetic inhibition of PVT^Vglut2-^insular cortex projections during stress-induced binge eating

Mice (*n* = 40) underwent the stress-binge protocol as described above. On test day, we injected mice with either vehicle (saline, tdT (*n* = 10) and hM4Di (*n* = 10)) or DREADD ligand clozapine-N-oxide (CNO; tdT (*n* = 10) and hM4Di *n* = 10) into the insula immediately prior to the behavioral task. Animals were exposed to the stressor on days 8, 16, whereby all mice were micro-infused with vehicle, and the final stress-binge test was performed on day 24, whereby half of the mice received vehicle (5% DMSO in 0.9% saline) and the other half CNO (1 mM in vehicle; In Vitro Technologies, Australia) micro-infusions in a randomized order [27]. Bilateral micro-infusions (500 nL/hemisphere) were performed over 2 min immediately prior to behavioral testing. Time spent interacting with the palatable food container, latency to start eating, and time spent eating were measured by manual scoring of video recordings by a blinded experimenter. Following this, we examined locomotor activity (15 min habituation followed by micro-infusion of CNO or vehicle then 10 min of locomotor activity monitoring in activity chambers), anxiety-like behavior using the light/dark box (CNO/vehicle infusion immediately followed by 10 min light/dark test), and palatable food consumption independent of stress (CNO/vehicle infusion in home-cage followed by measurement of palatable food consumption, 2 h).

### Immunohistochemistry

1-in-4 series of whole brain was processed for immunohistochemical detection of Fos-protein and GFP or mCherry. See supplemental information for details.

### Multiplex fluorescent in situ hybridization

RNAscope was used to detect Vglut2 (*Slc17a6*; 319171-C3) and Cre (312281-C2) in PVT to determine validity of Cre expression *Vglut2*-Cre mice (*n* = 3, Fig. S1).

### Statistical analysis

For full statistical methods please see supplemental information. Statistical analyses were performed using GraphPad Prism Version 8 (GraphPad software Inc; USA) and Python (version 3.8). Data are expressed as mean  ±  *SEM* and were considered significant at *p* < 0.05.

## Results

### Stressed animals display binge-like behavior and overconsume palatable food

First, we confirmed our previous observation that an “emotional stressor” promotes binge-like eating in female mice. Mice subjected to a stressor displayed pronounced binge-like behavior on test day, consuming the equivalent of 38% of their usual daily chow intake, in just 15 min (Fig. 1B; unpaired t-test, *p* = 0.0019).  We also confirmed that exposure to the tea strainer induces stress. Activation of the paraventricular nucleus of the hypothalamus (PVN) was observed in response to this stressor; and differences in Fos protein expression between groups was observed (F_(3,16)_ = 22.17, *p* < 0.0001, One-way ANOVA; Fig. 1D). For full statistics refer to table S2. Plasma corticosterone levels were also elevated in mice from the stress only group (Fig. 1E). Ordinary one-way ANOVA revealed differences in corticosterone expression across different groups (*F*_(2,16)_ = 6.780, *p* = 0.0074). Increased corticosterone levels were observed in plasma from mice subjected to the stress manipulation when compared to control group (*p* = 0.0178). Mice from stress-binge group had plasma corticosterone levels similar to controls and significantly less than the stress only group (*p* = 0.0148).

### Stress-induced binge eating alters neural co-activation patterns

Fos mapping throughout the neuroaxis revealed patterns of activation distinct to control, stress only and stress-binge conditions (full results Table S1, Fig. S2, S3). Each module contains brain regions with a similar pattern of co-activation and is suggestive of enhanced functional connectivity between the brain regions in that module. The bigger the size of the module the more brain regions are involved with similar patterns of co-activation. The number of modules reflects the general inter-regional co-ordination of activation. The particular closeness in similarity/extent of coactivation between two particular regions is the Euclidean distance (highly correlated coactivation between two brain regions = small distance/warm color; uncorrelated pattern between two groups = large distance/cool color).

**Fig. 2:**
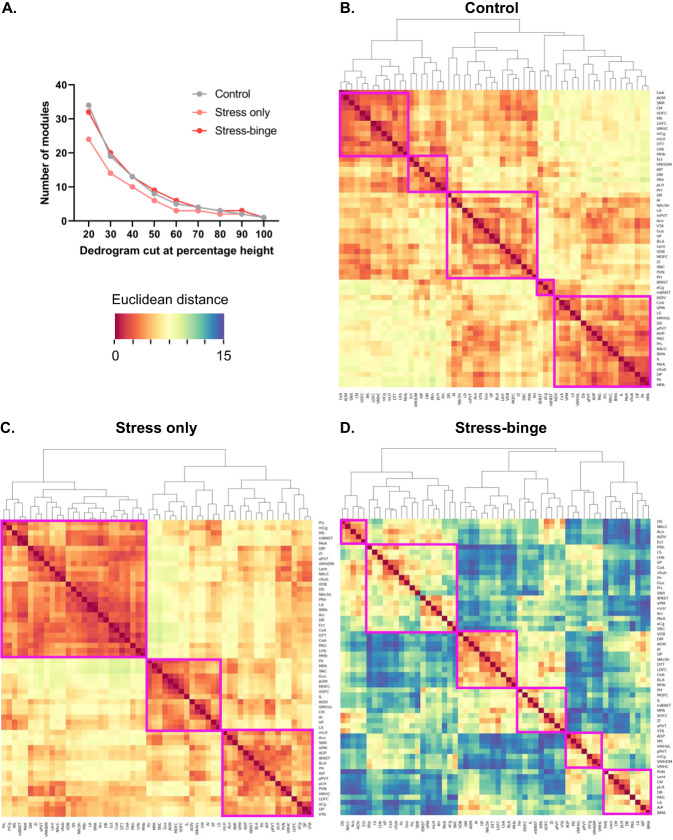
Hierarchical clustering of Euclidean distance matrices for control, stress only and stress-binge groups. Modules were determined by cutting each dendrogram at 60% of the maximal height and are identified by magenta rectangles. **A** Number of modules for each group after trimming the hierarchically organized dendrogram at different percentages of the tree height. **B** Corresponding Euclidean distance of each brain region relative to the others in control mice. Five modules of coactivation were delineated. **C** Corresponding distance of each brain region relative to the others that were examined in stress only mice. Three modules of co-activation were delineated. **D** Corresponding distance of each brain region relative to the others that were examined in stress-binge mice. Six modules of co-activation were delineated. ACo Anterior cortical amygdaloid nucleus, AI Anterior insula, AIDV Agranular insular cortex, dorsal-ventral subdivision aPVT, paraventricular nucleus of the thalamus, anterior part, Arc Arcuate hypothalamic nucleus, BLA Basolateral amygdala, BMA Basomedial amygdala, CeA Central nucleus of the amygdala, CM Central medial nucleus of the thalamus, cVMH Ventromedial hypothalamic nucleus, central division; CxA Cortex-amygdala transition zone, DM Dorsomedial hypothalamic nucleus, dmVMH Ventromedial hypothalamic nucleus, dorsomedial division, DP Dorsal peduncular cortex, DR Dorsal raphe, DS Dorsal striatum, DTT Dorsal tenia tecta; dvAI; Ect Ectorhinal cortex, IL Infralimbic cortex, LA Lateral amygdala, lBNST Bed nucleus of stria terminalis, lateral division, lEnt Lateral entorhinal cortex, lHb Lateral habenula, lOFC Orbitofrontal cortex, lateral subdivision, LS Lateral septum, MS Medial septum, mAON Anterior olfactory nucleus, medial part, mBNST Bed nucleus of the stria terminalis, medial division; mCg, cingulate cortex, medial part; MeA Medial nucleus of the amygdala, mHb Medial habenula, mLH Lateral hypothalamus, medial division; mOFC Orbitofrontal cortex, medial subdivision; mPOA, medial preoptic area; NAcC, nucleus accumbens core; NAcSh, nucleus accumbens shell; PAG, periaqueductal gray; pAI, agranular insular cortex, posterior part; pAON, anterior olfactory nucleus, posterior part; PH, posterior hypothalamic area; Pir Piriform cortex, pLH Lateral hypothalamus, posterior division; pPVT, paraventricular thalamus, posterior part; PRh, perirhinal cortex; PrL, prelimbic cortex; PVN, paraventricular nucleus of the hypothalamus, medial parvicellular part; SNc Substantia nigra compacta; SNr, substantia nigra reticulata; VDB, nucleus of the vertical limb of the diagonal band; vlVMH, ventromedial hypothalamic nucleus, ventrolateral division; vOFC, orbitofrontal cortex, ventral subdivision; VP, ventral pallidum; VPM, ventral posteromedial thalamic nucleus; VPMpc, ventral posteromedial thalamic nucleus, parvicellular portion; vSub, ventral subiculum; VTA, ventral tegmental area; ZI, zona incerta.

Hierarchical clustering identified a distinct modular organization of brain activation patterns in response to palatable food consumption (control), exposure to the stressor only (stress only) and stress-bingeing behavior (stress and palatable food). Coactivation patterns in the brains of control mice (Fig. 2B) were organized into five modules, where regions within these modules showed more synchronous coactivation patterns (i.e. warmer colors in heatmap, Fig. 2B). By contrast, stress only brains (Fig. 2C) showed decreased modularity compared to control brains, being organized into three large clusters. Across different clustering levels, the stress-only condition consistently showed a lower number of modules compared to both control and stress-binge brains (Fig. 2A). Like control brains, however, coactivation patterns in the stress only brains were also highly correlated (hence both Fig. 2B, C show primarily warm colors). In contrast, a reduction in this synchrony is observed in the stress-binge group, demonstrated by the flip from warm to cool colors in the heatmap (Fig. 2D). Further, a greater number of modules is observed (Fig. 2D), demonstrating a disparate pattern of co-activation during this behavior. Lastly, these 6 modules were also smaller in size overall, reflecting the smaller number of brain regions that showed synchrony in their activation patterns in the stress-binge condition.

The first module in stress only brains (top left, Fig. 2C) primarily consisted of mesocorticolimbic areas, including the NAc, subdivisions of the amygdala, cingulate and prelimbic cortices, and had slightly opposing activation patterns compared to cluster two (middle), which largely comprised areas involved in stress responses, such as medial preoptic area and lateral septum, as well as gustatory regions. The third cluster (bottom right), had similar activation patterns to cluster two and primarily comprised hypothalamic areas, including the PVN, LH, central VMH, and posterior hypothalamus.

The largest module identified in stress-binge brains (Fig. 2D) comprised a mix of limbic, hypothalamic, and thalamic regions, including the ventral pallidum, lateral BNST, medial LH, arcuate nucleus, VPM and VPMpc. These regions had opposing activation patterns when compared to the third module and included many corticolimbic regions such as the anterior insular cortex, dorsal peduncular area, NAcSh, central amygdala and basolateral amygdala. Of note, regions of the largest cluster in stress only brains, including the anterior PVT, had opposing Fos activation patterns and less activation in stress-binge brains, suggesting a shift in their functional roles once animals were allowed to binge. Similarly, in the stress only brains, regions that were highly activated and coupled together in a cluster, such as the posterior insular cortex, PVN, central VMH, and posterior PVT, were part of opposing modules in stress-binge brains. These regions became more distantly connected, representing a shift in their activation pattern, which suggests a weaker relationship between these regions in stress-binge animals as compared to stress only animals. Collectively, these data provide insight into changes in connectivity which underpins stress-induced bingeing. Next, we wanted to determine a role for specific components of this network in the behavior.

### PVT^Vglut2+^ projections to the insular cortex are activated following stress-induced bingeing

**Fig. 3:**
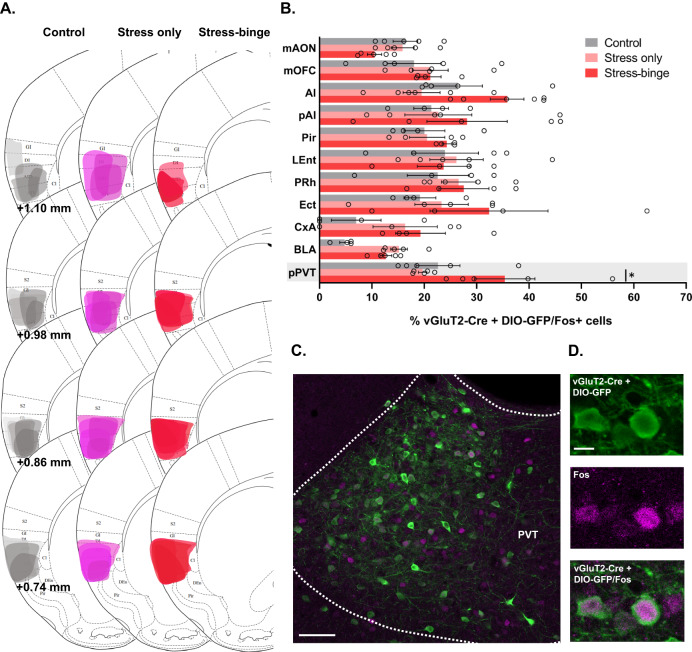
Insular cortex^Vglut2+^ afferents co-localize with Fos protein expression during stress-induced binge eating protocol. **A** Representative plots of the spread of the retrograde virus in the injection site for the three groups. **B** Brain regions with the highest number of Fos-positive Vglut2 projecting neurons to the insular cortex. Increased Fos expression in PVT^Vglut2^ projecting neurons was observed in animals that stress-binged and is highlighted in grey. *p = 0.0308, two-way ANOVA followed by Tukey’s multiple comparisons test. Data are presented as mean ± SEM, *n* = 4–6 mice per group. **C** Photomicrograph of the PVT showing Vglut2-Cre projecting neurons (green) and Fos-positive cells nuclei (magenta). Scale bar: 100 μm. **D** Representative image of PVT immunofluorescence for Vglut2 (green), Fos-protein (magenta), and merged fluorescence. Scale bar: 20 μm. Ai Anterior agranular insular cortex, BLA Basolateral amygdala, CxA Cortex-amygdala transition zone, Ect Ectorhinal cortex, LEnt Lateral entorhinal cortex, mAON Anterior olfactory nucleus, medial portion; mOFC Orbitofrontal cortex, medial portion, PRh Perirhinal cortex, PVT Paraventricular nucleus of the thalamus, pPVT Paraventricular nucleus of the thalamus, posterior portion.

Of all the brain regions that had increased Fos expression following stress-induced binge eating, only the insular cortex showed a positive correlation between the extent of Fos immunolabelling and bingeing behavior (Fig. S3D). Based on this we investigated glutamatergic projections to the insular cortex using a combination of retrograde tracing and Fos immunohistochemistry. A Cre-dependent retrograde tracer was injected into the insular cortex of Vglut2-Cre mice in order to label Vglut2 projections to the insular cortex. Ipsilateral counting of double labelled cells (tracer + Fos) revealed a number of brain regions which exhibited moderate to high number of Fos-positive Vglut2 projecting cells to the insular cortex (Fig. 3A). Two-way ANOVA revealed a main effect of group (*F*_*(2,119)*_ = 5.168, *p* = 0.0070) and brain region innervation (F_*(10,119)*_ = 4.753, *p* < *0.0001)*. Strong glutamatergic inputs from the PVT were observed of which 35% were also positive for Fos-protein in stress-binge animals. This high overlap of Vglut2 and Fos-protein was significantly higher in the stress-binge group only in the PVT (*p* = 0.0308), suggesting that a Vglut2+ pathway from PVT to insula may mediate this behavior.

### PVT^Vglut2+^ input to the insular cortex regulates stress-induced binge eating

**Fig. 4:**
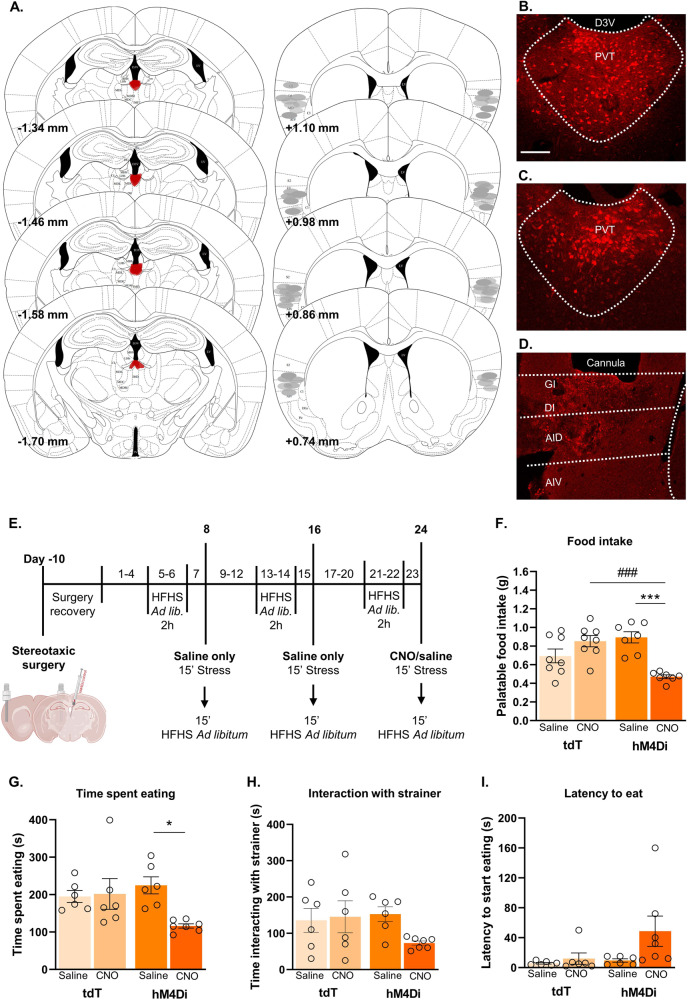
Inhibition of PVT^Vglut2+^-insular cortex projections attenuates stress-induced binge eating. **A** Inhibitory DREADD injection into the PVT and viral spread (mm from bregma; left) and approximate cannula placements of the injector tips for the insular cortex (right). Representative photomicrographs of Cre-dependent control virus (**B**) and Cre-dependent hM4Di virus (**C**) spread in the PVT. **D** Representative photomicrograph of cannula placement in the insular cortex. **E** Behavioral testing timeline for chemogenetic manipulations. After stereotaxic surgery for cannulae placement in the insular and viral delivery into the PVT, mice were subjected to the stress protocol, locomotor activity test, food consumption independent of stress test, and light/dark box transition test. **F** CNO administration significantly reduced the amount of palatable food consumed by hM4Di-expressing mice (main effect of treatment *F*_(1,26)_ = 5.043, *p* = 0.0335; interaction *F*_(1,26)_ = 24.34, *p* < 0.0001; hM4Di CNO vs hM4Di saline *p* = 0.0002, hM4Di CNO vs tdT CNO *p* = 0.0005). A trend was observed in hM4Di CNO vs tdT saline groups, *p* = 0.056. **G** hM4Di CNO treated animals spent less time consuming the palatable food post-stress (main effect of treatment, *F*_(1,21)_ = 0.488, *p* = 0.0462; interaction, *F*_(1,21)_ = 5.704, *p* = 0.0264; *p*osthoc comparison, *p* = 0.0229 vs hM4Di saline). **H** No significant differences were seen in time spent interacting with the tea strainer between groups. **I** No significant differences were seen between groups in the latency to start consuming palatable food once available post-stress. *p < 0.05; ***,^###^*p* < 0.005, two-way ANOVA followed by Tukey or Sidak’s multiple comparisons test when applicable. Data = mean ± *SEM*. tdT saline, *n* = 6–8; tdT CNO, *n* = 6–8; hM4Di saline, *n* = 6–7; hM4Di CNO, *n* = 7. CNO, clozapine-N-oxide; tdT, tdTomato.

To determine whether stress-induced binge eating requires PVT^Vglut2^-insular cortex projections, we chemogenetically inhibited this pathway using an hM4Di DREADD (injected into PVT, CNO microinfused into insula on test day). Chemogenetic inhibition of this pathway reduced binge eating of the palatable food (*p* < 0.001 Fig. 4F) and time spent eating during the binge episode (*p* = 0.0229, Fig. 4G) compared to control groups. In contrast, chemogenetic inhibition had no effect on the time spent interacting with the tea strainer containing the palatable food during the stress period, nor latency to start consuming the food (Fig. 4H, I). Collectively, these results demonstrate a causal role for PVT^Vglut2^-insula circuit in mediating bingeing as a coping mechanism following stress.

### Inhibition of PVT^Vglut2+^-insular cortex projections does not affect locomotor activity

**Fig. 5:**
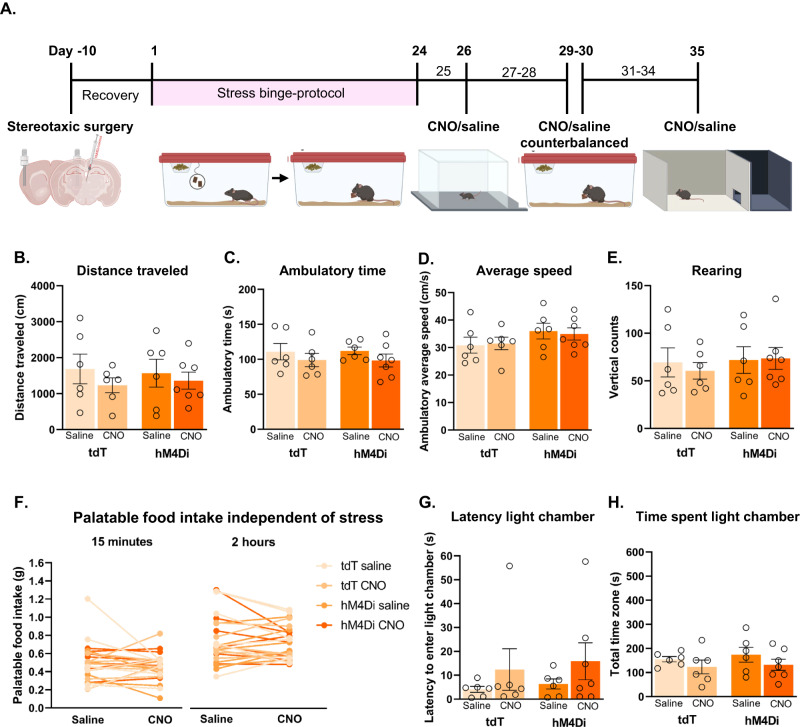
Pathway-specific inhibition of PVTV^glut2+^-insular cortex projections does not impact locomotor activity, anxiety-like behavior or hedonic eating independent of stress. **A** Behavioral testing timeline. Locomotor activity was tested on day 26 following infusion of saline or CNO. Palatable food consumption independent of stress was assessed on days 29 and 30 and CNO and saline were delivered across these 2 days in a counterbalanced manner. Light-dark box test was performed on the final day of testing. For locomotor activity, data presented as (**B**) total distance travelled (cm) (**C**) ambulatory time (s), (**D**) ambulatory average speed (cm/s), and (**E**) number of rearing episodes during locomotor testing period (10 min). **F** Palatable food consumption independent of stress. No main effect of CNO or activation of hM4Di DREADD was seen under non-stress conditions (two-way RM ANOVA). Data for the light dark/box test as follows: **G** Latency to enter the light compartment (s). **H** Total time spent (s) in light compartments. tdT saline *n* = 6, tdT CNO *n* = 6, hM4Di saline *n* = 6, hM4Di CNO *n* = 7. Data presented as mean ± SEM.

To determine whether chemogenetic inhibition of binge eating was due to a general sedating effect, we examined the impact of hM4Di inhibition on locomotor activity. Locomotor activity was not affected by inhibition of PVT^Vglut2^-insula neurons or micro-infusions of saline or CNO (Fig. 5B–E).

### Inhibition of PVT^Vglut2+^-insular cortex projections does not impact hedonic eating in the absence of stress

We next determined whether chemogenetic inhibition of binge eating was due to a general reduction in palatable food consumption (i.e. under no stress conditions). To this end, we measured palatable food consumption in the home cage without the presence of stressful manipulation. Counterbalanced delivery of saline or CNO had no impact on palatable food consumption at both the 15 min and 2 h time points (Fig. 5F). This data shows that inhibition of PVT^Vglut2^-insular cortex projections does not impact hedonic eating in the absence of stress.

### PVT^Vglut2+^-insular projections do not modulate anxiety-like behavior

Given PVT is a key node in regulation of anxiety-like behavior and some sex differences have been in observed in this regard [28], we investigated the impact of chemogenetic inhibition this behavior. We used the light-dark test to assess the impact of hM4Di inhibition on baseline anxiety-like behavior (Fig. 5G–H). Neither micro-infusions of saline nor CNO affected latency to enter the light compartment (Fig. 5G) or time spent in light compartment (Fig. 5H), supporting our hypothesis that PVT^Vglut2+^-insular projections do not modulate anxiety-like behavior.

## Discussion

We set out to determine the neural underpinnings of ‘emotional’ stress driven eating in females. We report here three main findings. First, we observed that co-activation patterns across the mouse brain are altered by stress-induced binge eating.  Second, stress-induced binge eating in female mice was associated with increased Fos activation of Vglut2 PVT cells projecting to the insula. Lastly, chemogenetic inhibition of this projection reduced stress-induced binge eating, without affecting locomotor activity or anxiety-like behavior. These data demonstrate a causal role for this PVT^Vglut2+^-insular cortex projection in bingeing induced by ‘emotional’ stress in females.

### Validation of stressor

We confirmed that the manipulation used to precipitate bingeing in our model induces stress. Elevated corticosterone and PVN activation in mice subjected to stress without subsequent access to palatable food is evidence of HPA axis activation. This mirrors findings of elevated corticosterone in a model using a similar type of stressor in rats [29]. The stimulus used in our protocol likely represents a ‘frustrative stressor’ to the mice, however it could also simply be acting as a powerful olfactory and visual ‘prime’ which precipitates binge eating. Either way, a robust binge is observed, over and above control levels, and does not require a history of caloric restriction; thus demonstrating the utility of this mouse model.

In line with the concept of “comfort-eating”, subsequent access to palatable food in our study reduces both corticosterone levels and PVN activation to control levels. This is consistent with the observations that PVN activity in rodents and cortisol levels in humans can be suppressed by consumption of a palatable food reward [30, 31], supporting the idea that palatable foods are used as means to regulate negative emotional states [32, 33].

### A distinct pattern of neural co-activation underlies stress-induced binge eating

Examination of co-activation patterns revealed differences between control, stress only and stress-binge conditions. Both control and stress only conditions showed strong synchrony in patterns of co-activation throughout the brain. This was particularly apparent in the stress only condition given brain regions were consolidated into three large modules. A similar increase in synchrony/decrease in modularity has been observed in response to social defeat stress [34], as well as during abstinence from long term alcohol use [35], a state known to be associated with dysregulated HPA axis function [36]. Given that the stress response can be considered an ‘emergency state’ for an organism [37], it follows that the brain would be working together in a more coordinated and synchronous fashion in response to the perceived threat. By contrast, a lack of synchrony and interregional co-activation is observed in response to the bingeing behavior following stress. Indeed, very few brain regions show correlated co-activation patterns, and, in fact, most are anti-correlated (blue in heatmap). This suggests the experience of stress-induced binge eating may reorganize co-activation networks. This is in contrast to mice who consumed palatable food in the absence of stress exposure, suggesting it is the unique condition of bingeing following stress that is associated with a distinct pattern of asynchrony across the brain.

Though no other hierarchical clustering studies have been performed in animal models of binge eating, one resting state fMRI study used graph theory to characterize functional brain networks of women with bulimia nervosa as compared to healthy controls and found altered intrinsic functional brain architecture [38]. Specifically, hypoconnectivity was observed involving subcortical (striatum, thalamus, insula, amygdala, putamen), and paralimbic (orbitofrontal cortex, parahippocampal gyrus) regions, consistent with the lack of synchrony in coactivation observed between these brain regions in our study.

### PVT^Vglut2+^-insular cortex projections and stress-induced binge eating behavior

Fos data strongly suggested that a PVT^Vglut2+^-insular cortex projection is activated during stress-induced binge eating in our mouse model. This is consistent with the established role for PVT in both reward seeking behavior [39–41] and the response to stress [42] PVT neurons are activated by palatable food [43], contexts/cues associated with reward [44–47], drug-seeking behavior in reinstatement paradigms [46–48] as well as in response to both acute [49–51] and chronic stressors [52].

Our chemogenetic experiments causally implicated a PVT^Vglut2^-insular cortex projection in stress-induced binge eating. A trend towards an increased latency to eat palatable food after the stressor was also noted in hM4Di CNO treated animals. These data are consistent with the notion of “comfort-eating”, where palatable foods are used as means to regulate negative emotion [38, 53, 54]. These findings are also consistent with previous work describing a role for PVT in mediating hedonic overconsumption of natural rewards as well as both food- and drug-seeking behavior [55]; however, to date, there has been relatively scant exploration of PVT in stress-driven reward seeking behavior specifically. Blockade of PVT orexin1 receptors has been shown to prevent stress-induced reward seeking for sweetened condensed milk [56], implicating PVT orexin signaling through orexin 1 receptor in stress-driven eating. The LH sends a strong orexinergic projection to PVT and is a possible source of input to our insular cortex projecting PVT^Vglut2^ cells. Other possible inputs include other hypothalamic regions, brainstem inputs, prefrontal cortex and ZI [57].

Elegant work has recently described a dichotomy between GABAergic input to PVT from LH versus glutamatergic input from PFC in coding the consummatory aspect of sucrose consumption versus the cue-reward association [58]. However, it is unlikely these PVT inputs are also involved in stress-driven eating in females, as both inputs synapse onto NAc-projecting PVT neurons, whereas our findings implicate insula-projecting PVT neurons. These NAc-projecting PVT cells have also been specifically implicated in sucrose- but not saccharin-seeking behavior [58], sucrose-seeking during reward omission [59], high-fat feeding [60], and opiate withdrawal [61]. By contrast, the functional role of insula-projecting PVT cells remains understudied. To our knowledge this study is the first to implicate a PVT to insular cortex projection in any model of food overconsumption.

Importantly, our data shows selective involvement for a PVT^Vglut2^-insular cortex projection in stress-eating specifically and not in mediating hedonic eating per se. In this sense, despite extensive literature describing the role of PVT (particularly the projection to NAc) in mediating hedonic eating, it is this specific insular pathway that appears to be driving eating precipitated by stress.

Given stress-driven bingeing is only displayed by females in our model it is not possible to test sex-specific involvement of the PVT^Vglut2^-insular cortex projection in this behavior. Nevertheless, relevant evidence exists describing sex differences in these two brain regions. One recent study showed activation of PVT in response to hedonic feeding in sated females but not males. Of note, the same sex difference was not observed in insula [62]. Further, stress-induced electrophysiological changes in PVT neurons are observed in males but not females, providing a possible mechanism underlying the impairment in habituation to restraint stress observed in females [63]. Stress also differentially influences emotional perception in females versus males in relation to insula connectivity [64]. Thus, it could be that in females stress alters thalamic coding of food-related information, potentially leading to enhanced food motivation, surpassing homeostatic states and influencing the processing of interoceptive signals (i.e. emotions, stress) by the insula.

In summary, we show that PVT^Vglut2+^ cells projecting to insula are activated during stress-induced binge eating and this projection is essential for expression of this behavior in females. The PVT is a key hub for reward processing that integrates information related to internal states with environmental stimuli to orchestrate motivated behaviors. It is possible that the connectivity between the insula and PVT becomes dysfunctional following stress, leading to aberrant processing of internal states and food palatability. This may lead to the emergence of maladaptive behaviors such as binge-like eating. This study provides the novel insight into the precise neural circuits specifically governing ‘emotional’ stress induced binge eating in females.

### Supplementary information

Supplemental Material
