# Supplementary material for: A paraventricular thalamus to insular cortex glutamatergic projection gates “emotional” stress-induced binge eating in females
Source: Neuropsychopharmacology. 2023 Jul 20;48(13):1931–40. doi: 10.1038/s41386-023-01665-6 (PMC10584903; doi:10.1038/s41386-023-01665-6)
Supplement: Supplementary file 1 — Supplemental Material [file 41386_2023_1665_MOESM1_ESM.docx]

**Supplemental Material**

**Supplemental methods**

**Stress-induced binge eating protocol**

The stress-binge protocol is comprised of multiple 8-day cycles. During each 8-day cycle, on day 5 and 6 mice are given 2h access to highly palatable food rich in fat and sugar (Reese's® peanut butter drops and Nestlé® chocolate drops) at the beginning of the dark cycle (0900-1100). *Ad libitum* standard chow is available throughout the entire experiment. On day 8 mice are exposed to an ‘emotional’ stressor. The stressor consists of an enclosed tea strainer containing palatable food placed within the home cage. Mice have visual and olfactory access to the food but could not consume it for 15min. Following this, the palatable food is placed in the home cage of ‘stress-binge’ mice, who are allowed to freely consume the food. The amount of palatable food consumed is monitored over a 15min period. For the control group, mice have access to the palatable food for 15min but do not experience the stressor. For the Fos/tracer dual labelling experiment 2 x 8-day cycles were performed. For the chemogenetic experiment 3 x 8-day cycles were performed as mice needed time to habituate to the microinfusion procedure.

For the Fos/tracer dual labelling experiment, “stress only” mice were not provided palatable food following exposure to the stressor. Naïve animals were not subjected to the behavioral paradigm nor received any manipulation but were housed in the same room over the same experimental timeframe.

**Serum collection and corticosterone quantification**

Following the last behavioral timepoint, animals were euthanized and blood (~200µl) was collected via cardiac puncture with ethylenediaminetetraacetic acid (EDTA; 0.1M) coated syringes and placed in specialized collection tubes (MiniCollect 0.25/0.5mL K3 EDTA; Greiner Bio-One, Kremsmunster, Austria) for corticosterone analysis. Blood was allowed to clot at room temperature for 30min then centrifuged for 10min at 1000 RCF (4°). Serum was transferred to fresh tubes and stored at -20° until further use. Corticosterone levels were determined in duplicates by enzyme immunoassay corticosterone kit (Cayman Chemical, Michigan, US) following manufacturer’s instructions. Serum samples were diluted 1:100 microplate reader was set at 412nm. Corticosterone levels from control mice were used as baseline levels.

**Identification of coactivation of brain regions**

Inter-regional Pearson correlations were calculated by comparing the mean of Fos expression per mm^2^ between every region analyzed in the control (*n* =4-7 mice), stress only (*n*=5-6 mice), and stress-binge (*n*=5-8 mice) groups. Brain regions were organized following the Mouse Brain Atlas [1] based on their anatomical groups (olfactory system, cortex, basal ganglia and basal forebrain, hypothalamus, thalamus, and hindbrain) and bregma levels. A heatmap was generated for the purpose of visualizing possible clusters of coactivation.

**Surgical procedures and viral vectors**

We performed all surgeries a minimum of 10 days prior to the stress-induced binge procedure. We anaesthetized mice with isoflurane (5% induction, 1-2% maintenance) and placed them in a stereotaxic frame (Stoelting Co., Illinois, USA) for intracranial injections or cannulation surgery. Following surgery, analgesia (Meloxicam; 1 mg/kg, i.p., Troy Laboratories, AUS) and antibiotics (Baytril, enrofloxacin, 1 mg/kg, i.p, Bayer Health Care, AUS) were administered for 3 days.

**Viral spread and cannula placement validation**

We verified injection sites and cannula placements by delineating the insular cortex and PVT using the Mouse Brain Atlas coordinates [1]. We quantified starter cells and dense fluorescence from the retrograde virus, hM4Di DREADD or tdT control virus (8, 9). Injection sites of the retrograde virus in the insular cortex ranged from Bregma +0.62 to +0.98mm (anterior-posterior) and viral expression predominantly ranged from +1.18 to +0.38mm (anterior-posterior). We excluded brains that contained high viral spillover to surrounding regions. We considered injection sites where the spillover was minimal if no differences in inputs or outputs of the insular cortex were observed when compared to other injection sites that did not have any spillover. For injections into the PVT, we included brains in final analyses if AAV expression was contained to the PVT filling both hemispheres (primarily ranging from anterior-posterior -1.34 to -1.70 mm) and cannula placements in the insular cortex were in the correct coordinates, ranging from +1.10 to +0.38 mm relative to bregma.

**Fluorescent immunohistochemistry**

90min following the stress-induced binge eating task, mice were anesthetized with sodium pentobarbitone (80 mg/kg, i.p.; Virbac, Australia) and transcardially perfused with ∼10 mL of 0.1M Phosphate buffered saline (PBS, pH 7.4) followed by ∼30 mL of 4% w/v paraformaldehyde (PFA) in PBS. Brains were removed and postfixed in 4% PFA (1h), then transferred to PBS (1h), and finally transferred to 30% w/v sucrose in PBS (24h, 4°C). Brains were then frozen over dry ice and stored at -80°C until sectioning. Serial (40 μm) coronal sections were cut at -18°C by cryostat (Leica Microsystems, Germany) and sections stored in 0.1M PBS containing 0.1% sodium azide at 4°C.

**Fos-protein and GFP double labelling**

Immunohistochemical detection of Fos-protein and GFP was performed in 4 groups: stress-binge, stress only, control, naive. Sections were washed in PBS (3x10min) and incubated for 2h in 0.5% Triton-X,10% normal donkey serum (NDS) in PBS. Sections were then incubated 24h at 4°C with Fos primary antibody (#5348S, 1:1000; rabbit polyclonal anti-phospho c-Fos, Cell Signaling Technologies) and chicken anti-GFP primary antibody (#ab13970, 1:1000, Abcam) diluted in PBS triton-X with 2% NDS. Following primary incubation, sections were incubated for 2h in Alexa Fluor® 647-conjugated donkey anti-rabbit secondary antibody (1:600; Invitrogen) and Alexa Fluor® 488-conjugated donkey anti-chicken secondary (1:200, Jackson ImmunoResearch Laboratories) diluted in PBS triton-X with 2% NDS. All sections were then washed in PBS and mounted with DAKO fluorescence mounting medium (Agilent Technologies).

After image quantification (see below) we identified functional coactivation networks in stress-binge mice and compared them to networks from stress only and control mice. We then examined changes in modular structuring of the brain that are caused by stress-bingeing and identified key brain regions that may drive network function.

**mCherry immunoamplification**

For chemogenetic experiment, we performed immunoamplification of mCherry to visualize hM4Di expression in PVT and inputs to insula. Sections were immunolabeled as described above except with rabbit anti-dsRed primary antibody (1:1000, Clontech) followed by secondary antibody AF 594-conjugated donkey anti-rabbit (1:200, Invitrogen). TdTomato signal from control virus injections did not require immunoamplification for visualization.

**Image acquisition and neuronal quantification**

Investigators were blinded to experimental conditions during imaging and quantification. All brain coordinates were from Paxinos and Franklin Mouse Brain Atlas [1]. Images acquired using Zeiss Axio Observer LSM 780 confocal microscope (Zeiss, Oberkochen, Germany) with 20x and 40x objective magnification (1024 x 1024 magnification) and corresponding Zen Black software. The number of Fos and GFP-expressing neurons were quantified unilaterally (in the ipsilateral hemisphere in relation to injection site) across the brain. For insula quantification, sections adjacent to the injection site were considered. We manually quantified cells by first calculating the area size by outlining the brain regions, followed by manual counting of GFP-positive, Fos-positive and Fos+GFP-positive cells. We quantified all images using Zen Blue software (Zeiss, Germany). Data presented as mean Fos expression across two bregma levels per region per mm^2^.

**Locomotor activity**

Mice were habituated to the locomotor cell for 15min prior to test (*n*=6-7 per group) (27.3 cm (length) × 27.3 cm (width) × 20.3 cm (height), Activity Test Chamber #ENV-510; Med Associates inc). Following this, mice were micro-injected with either vehicle or CNO and locomotor activity (distance travelled, cm) was assessed for 10 min.

**Light/dark box**

We tested mice in automated locomotor cells (Activity Test Chamber #ENV-510; Med Associates inc) as previously published [2-3]. Each locomotor cell was equally divided into 2 distinct zones, a ‘light’ zone and a ‘dark’ zone. A small opening (10cm X 10cm) present in the midline wall of the ‘dark’ zone enabled mice to move freely between the 2 zones. The ‘light’ zone was lit by an array of light-emitting diodes (750 LUX) that were centrally placed. After infusing mice with either saline or CNO, animals (*n*=6-7 per group) were placed into the dark zone to begin the 10 min test. We automatically recorded the latency to enter the light compartment, total number of entries into the light compartment, and time spent in each compartment using Tru Scan 2.03 software (Coulbourn Instruments, USA). We performed testing during the animal’s light cycle, just before the transition period between light and dark cycles.

**Palatable food consumption independent of stress test**

We tested mice (*n*=6-7 per group) at the beginning of their dark cycle. Half of the animals received vehicle and the other half received CNO in their home cage on Day 29 of experimentation. On day 30, each mouse received the opposite micro-infusion as on Day 29 (counterbalanced). Fifteen min after the micro-infusion, we placed palatable food in the home cage and examined consumption 2h later. Standard chow was available during this task.

**Supplementary table S1. Bregma levels for Fos quantification across several brain regions.**





aCg, anterior cingulate cortex; ACo, anterior cortical amygdaloid nucleus; AI, anterior insula; aPVT, paraventricular nucleus of the thalamus, anterior part; Arc, arcuate hypothalamic nucleus; BLA, basolateral amygdala; BMA, basomedial amygdala; CeA, central nucleus of the amygdala; CM, central medial nucleus of the thalamus; cVMH, ventromedial hypothalamic nucleus, central division; CxA, cortex-amygdala transition zone; DM, dorsomedial hypothalamic nucleus; dmVMH, ventromedial hypothalamic nucleus, dorsomedial division; DP, dorsal peduncular cortex; DR, dorsal raphe; DS, dorsal striatum; DTT, dorsal tenia tecta; dvAI, agranular insular cortex, dorsal-ventral subdivision; Ect, ectorhinal cortex; EW, Edinger-Westphal nucleus; IL, infralimbic cortex; LA, lateral amygdala; lBNST, bed nucleus of stria terminalis, lateral division; lEnt, lateral entorhinal cortex; lHb, lateral habenula; lOFC, orbitofrontal cortex, lateral subdivision;  LS, lateral septum; MS, medial septum; mAON, anterior olfactory nucleus, medial part; mBNST, bed nucleus of the stria terminalis, medial division; mCg, cingulate cortex, medial part; MeA, medial nucleus of the amygdala; mHb, medial habenula; mLH, lateral hypothalamus, medial division; mOFC, orbitofrontal cortex, medial subdivision; mPOA, medial preoptic area; NAcC, nucleus accumbens core; NAcSh, nucleus accumbens shell; PAG, periaqueductal gray; pAI, agranular insular cortex, posterior part; pAON, anterior olfactory nucleus, posterior part; PH, posterior hypothalamic area; Pir, piriform cortex; pLH, lateral hypothalamus, posterior division; pPVT, paraventricular thalamus, posterior part; PRh, perirhinal cortex; PrL, prelimbic cortex; PVN, paraventricular nucleus of the hypothalamus, medial parvicellular part; SNc, substantia nigra compacta; SNr, substantia nigra reticulata;  VDB, nucleus of the vertical limb of the diagonal band; vlVMH, ventromedial hypothalamic nucleus, ventrolateral division; vOFC, orbitofrontal cortex, ventral subdivision; VP, ventral pallidum; VPM, ventral posteromedial thalamic nucleus; VPMpc, ventral posteromedial thalamic nucleus, parvicellular portion; vSub, ventral subiculum; VTA, ventral tegmental area; ZI, zona incerta. Bregma levels and abbreviations from Paxinos & Franklin, 2008 [1].

**Multiplex fluorescent *in situ* hybridization**

To determine validity of Cre expression in the PVT of the *Vglut2*-Cre mice we conducted Fluorescent in situ hybridisation. Adult male and female *Vglut2-*Cre mice (*n*=3, aged ~12 weeks) were briefly anaesthetized with isoflurane, euthanized by cervical dislocation, brains rapidly extracted and snap frozen in isopentane cooled on dry ice (Bacto Laboratories, NSW, Australia). Brains were stored at -80°C until use. Coronal sections of PVT were cut at 16μm in a 1/6 series using a cryostat (Leica Biosystems, NSW, Australia) and mounted directly onto Super Frost Plus slides (Fisher Scientific, NH, USA). Slides were stored at -80° C until use. The RNAscope Multiplex Fluorescent Reagent Kit (Advanced Cell Diagnostics, CA, USA) was used to detect Vglut2 and Cre in the PVT following the manufacturers protocol [5] and as previously described [6-7]. Briefly, slides were fixed in 4% w/v paraformaldehyde for 15min at 4°C, rinsed in 0.1M PBS (pH 7.4) followed by dehydration in ethanol (50%, 70%, 100%, and 100% v/v) for 5min each concentration. Slides were air-dried for 10 min at RT, and a hydrophobic barrier was drawn around each brain sections. Sections were then protease treated (pre-treatment 4) at room temperature for 15min. Next, sections were washed in distilled water followed by probe incubation. The target probes, Cre (312281-C2) and Vglut2 (*Slc17a6*; 319171-C3) were diluted in diluent and incubated at 40° C for 2 h. Following this, slides were incubated with amplifier probes (AMP1, 40°C for 30min; AMP2, 40° C for 15 min; AMP3, 40° C for 30 min and AMP4 AltB). This allowed detection of Cre in Alexa-488 and Vglut2 in Atto 647 channel. Finally, sections were incubated for 20s with DAPI and coverslipped with DAKO Fluorescent Mounting Medium (North Sydney, NSW, Australia).

A LSM 780 Zeiss Axio Imager 2 confocal laser scanning microscope (Carl Zeiss AG, Jena, Germany) using 40x and 63x objectives was used to take images of the PVT (pixel size 0.10µm), which were quantified from three sections per mouse (~Bregma -0.94 mm to -1.94 mm) using image J (National Institutes of Health; RRID:SCR_003070).

**Statistical analysis**

Unpaired t-tests were used to analyze the amount of palatable food consumed between 2 groups. Two-way ANOVA (factors: day and group) was used to analyze body weight, chow consumption, and palatable food consumption during intermittent access days. Tukey’s or Sidak’s multiple comparisons were utilized when applicable. Corticosterone data was analyzed by ordinary one-way ANOVA followed by Tukey’s posthoc. Immunohistochemical data analyzed as cell counts/mm^2^ for each bregma level depicted in supplementary table S1 (1 control mouse excluded for not eating any palatable food).

Fos-protein data were analyzed by one-way ANOVA followed by Tukey’s multiple comparisons or nonparametric version Kruskal-Wallis followed by Dunn’s post hoc where appropriate. 9 mice were excluded in total for various reasons (not eating any palatable food, not interacting with the food container during stress manipulation, off-target injections or low viral expression).

Inter-regional correlations of Fos/mm^2^ were determined using Pearson correlation analysis. Brain regions were defined according to the Mouse Brain Atlas (1). A heatmap was generated to visualize clusters of coactivation. Hierarchical clustering allows the visualization of modules that contain brain regions with a similar pattern of coactivation, based on Pearson’s correlations between 2 regions, and explores the functional connectivity between regions [2, 3]. Euclidean distances were then calculated using inter-regional Fos expression correlations between 2 brain regions. Regions were organized based on the distance using the complete linkage method to identify modules of coactivation [4]. Hierarchical clusters were trimmed at 60% dendrogram height. Dendrograms were created through complete linkage. Calculation of Euclidean distances and hierarchical clustering was performed using Python (version 3.8) in Jupiter notebook (version 6.3.0). Code can be found at <https://github.com/robertaanversa/>.

Amount of palatable food consumed, behavioral scoring, locomotor activity, and light/dark test data were analyzed by two-way ANOVA. Palatable food consumption independent of stress data were analyzed by two-way ANOVA and three-way ANOVA. 10 mice were excluded due to incorrect targeting. Statistical analyses were performed using GraphPad Prism Version 8 (GraphPad software Inc; USA) and Python (version 3.8). Data are expressed as mean ± *SEM* and were considered significant at *p*<0.05.

**Supplemental Results**

**Validation of the Vglut2-Cre mouse in the PVT**

*Vglut2*-Cre mice exhibited faithful expression of Cre in Vglut2-expressing neurons within the PVT, whereby 98.6% of Cre+ neurons co-expressed Vglut2 mRNA and 98.2% of Vglut2 neurons co-expressed Cre mRNA in the PVT (Fig. S2). These results show a minimal degree of ectopic Cre expression in the PVT and support the use of this mouse line in our study.

**
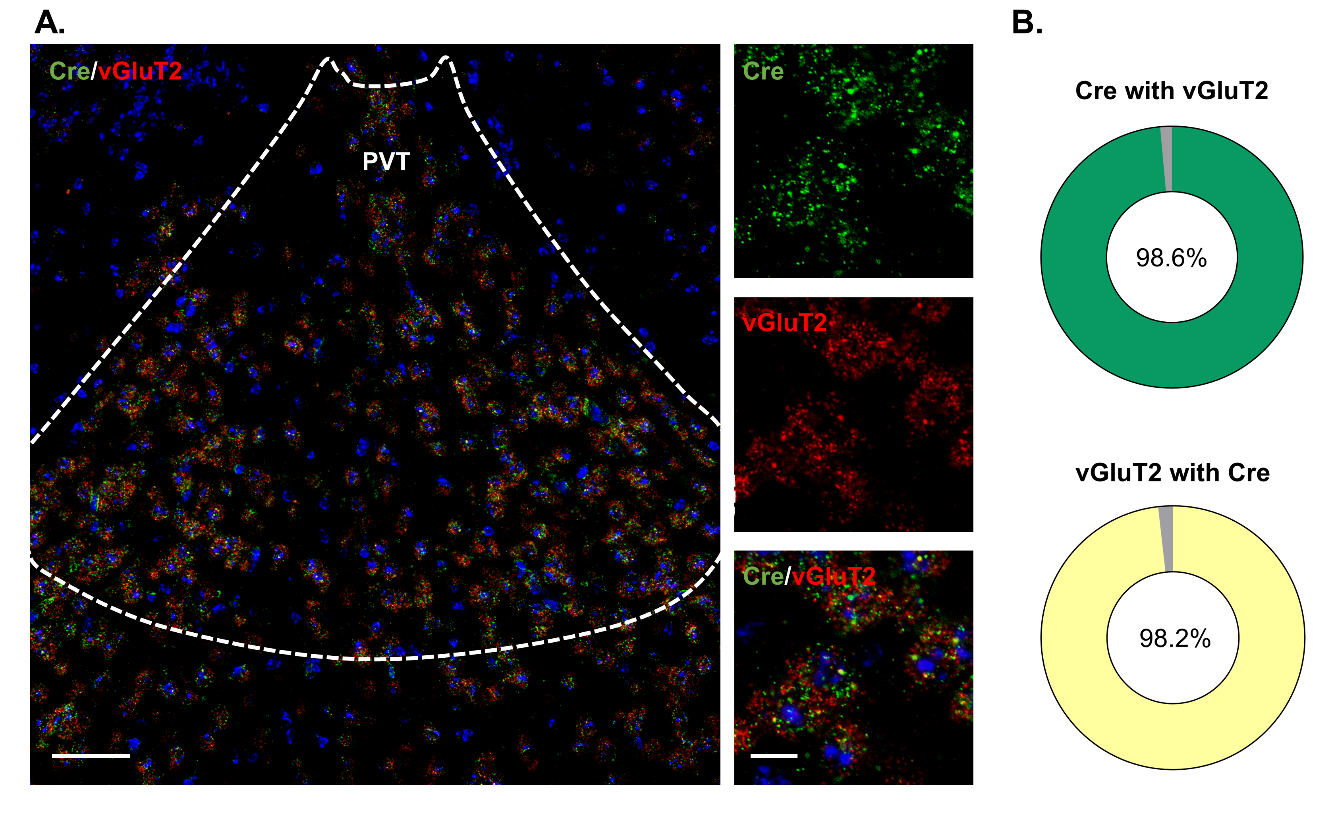
**

**Supplementary Figure S1. Cre and Vglut2 mRNA expression in PVT cells.** **A.** Overview and high magnification representative image of Cre, Vglut2 and overlay within PVT. **B.** Donut graph illustrating proportion of Cre expressing cells that also express Vglut2 and proportion of Vglut2 cells that co-express Cre. Co-expression of Vglut2 and Cre was higher than 98% in the PVT. Scale bars: 100μm (overview) and 20μm (high magnification). *n*=3 mice. PVT, paraventricular nucleus of the thalamus.

**Palatable food consumption independent of stress leads to distinct Fos-expression when compared to naïve animals**

In order to determine the pattern of brain activation distinct to palatable food consumption in the absence of stress, we compared Fos expression in the brains of this group to Fos expression in the brains of naïve animals. Significantly higher expression was observed in the anterior olfactory nucleus, medial and posterior parts, dorsal tenia tecta, orbitofrontal cortex, lateral, ventral and medial subdivisions, prelimbic cortex, anterior insula, anterior cingulate cortex, medial amygdala, medial preoptic area, PVN, medial portion of the lateral hypothalamus (mLH), arcuate hypothalamic nucleus, zona incerta, posterior hypothalamic area, medial habenula, anterior and posterior PVT, and ventral subiculum (*p*<0.05, one-way ANOVA with Tukey post hoc comparisons). Full results of statistical analyses are provided in supplementary table S2.


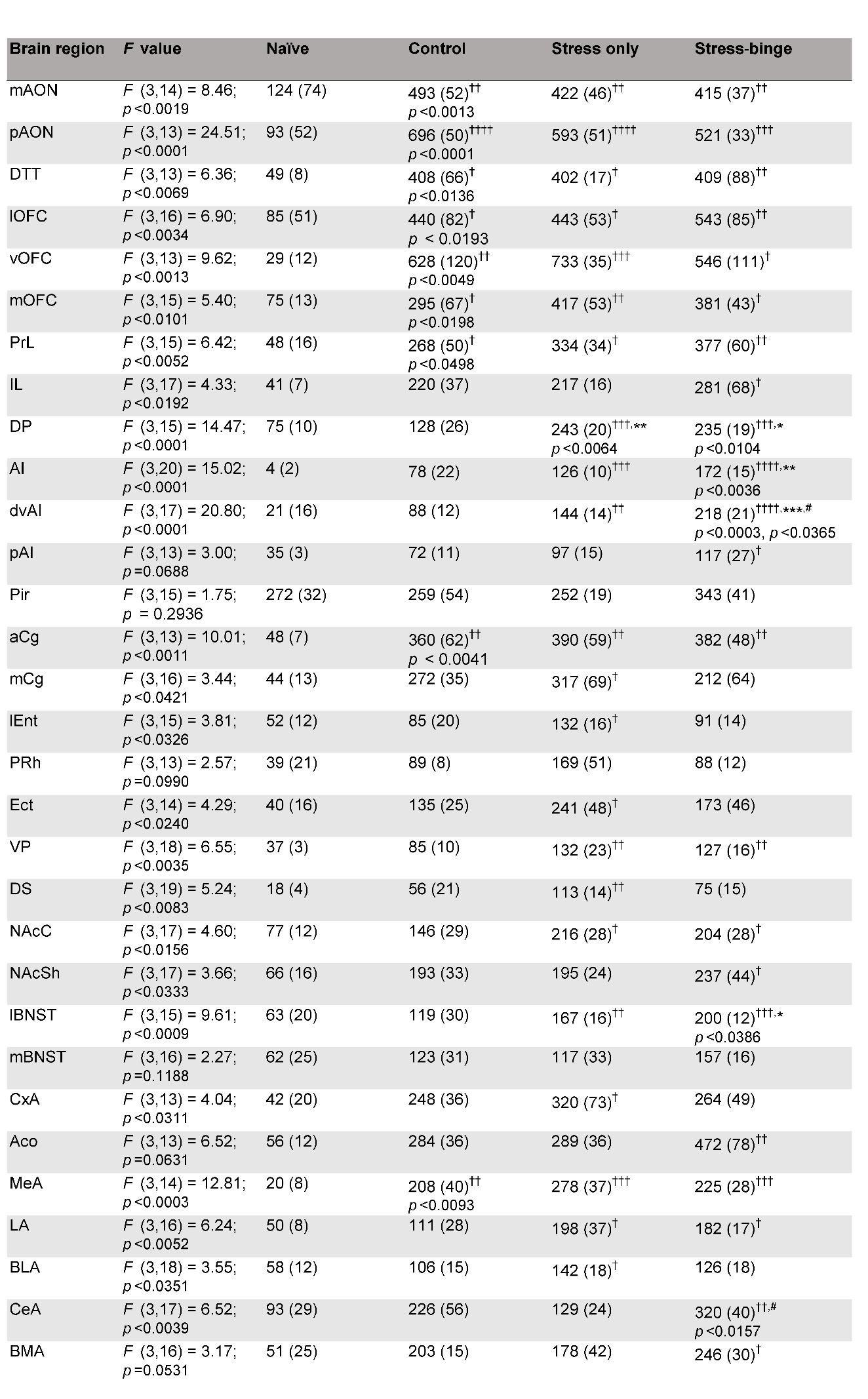
**Supplementary table S2. Fos-protein expression across several brain regions in naïve, palatable food consumption independent of stress (control), stress without access to highly palatable food (stress only), and highly palatable food consumption post-stress (stress-binge) groups.**


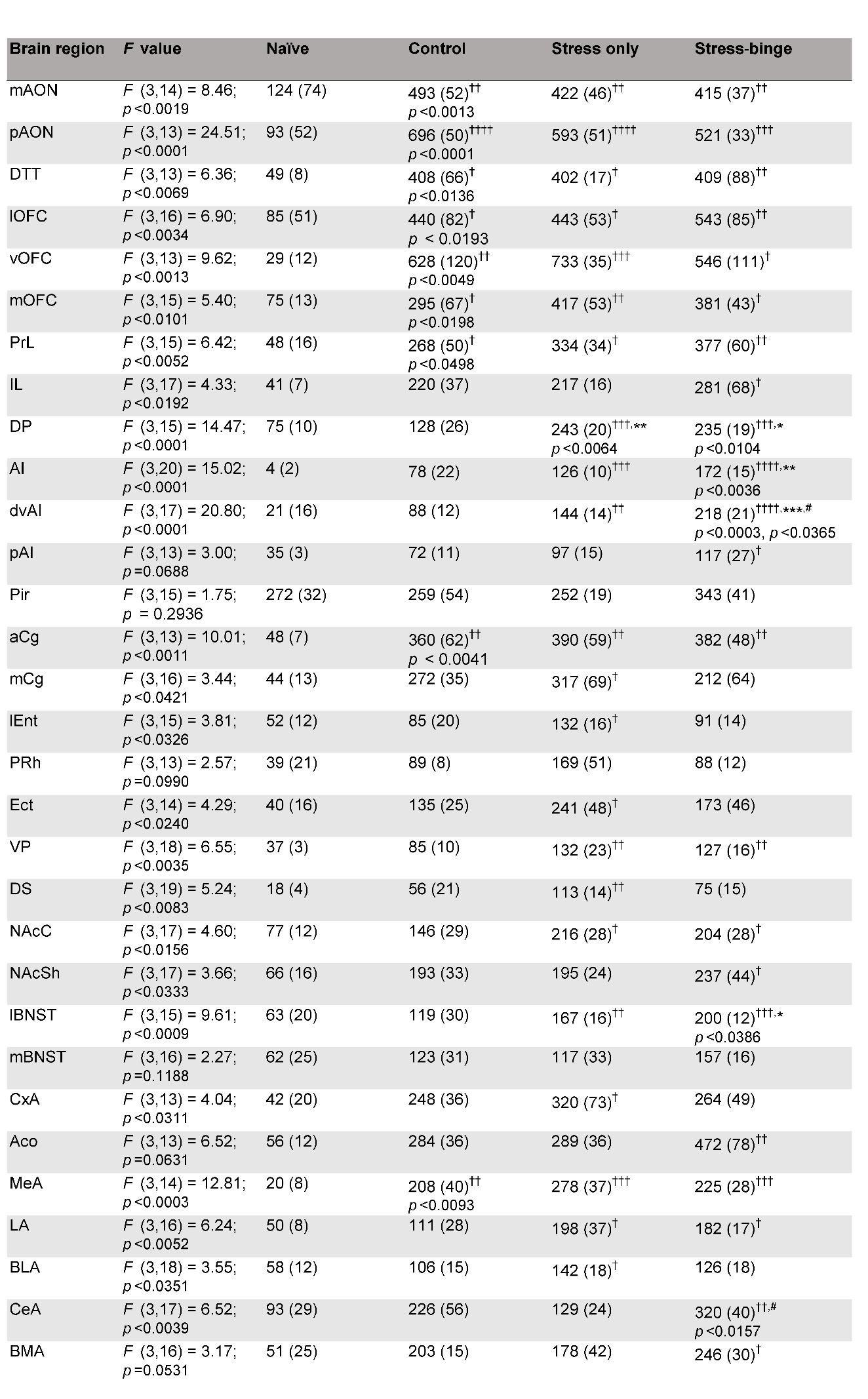


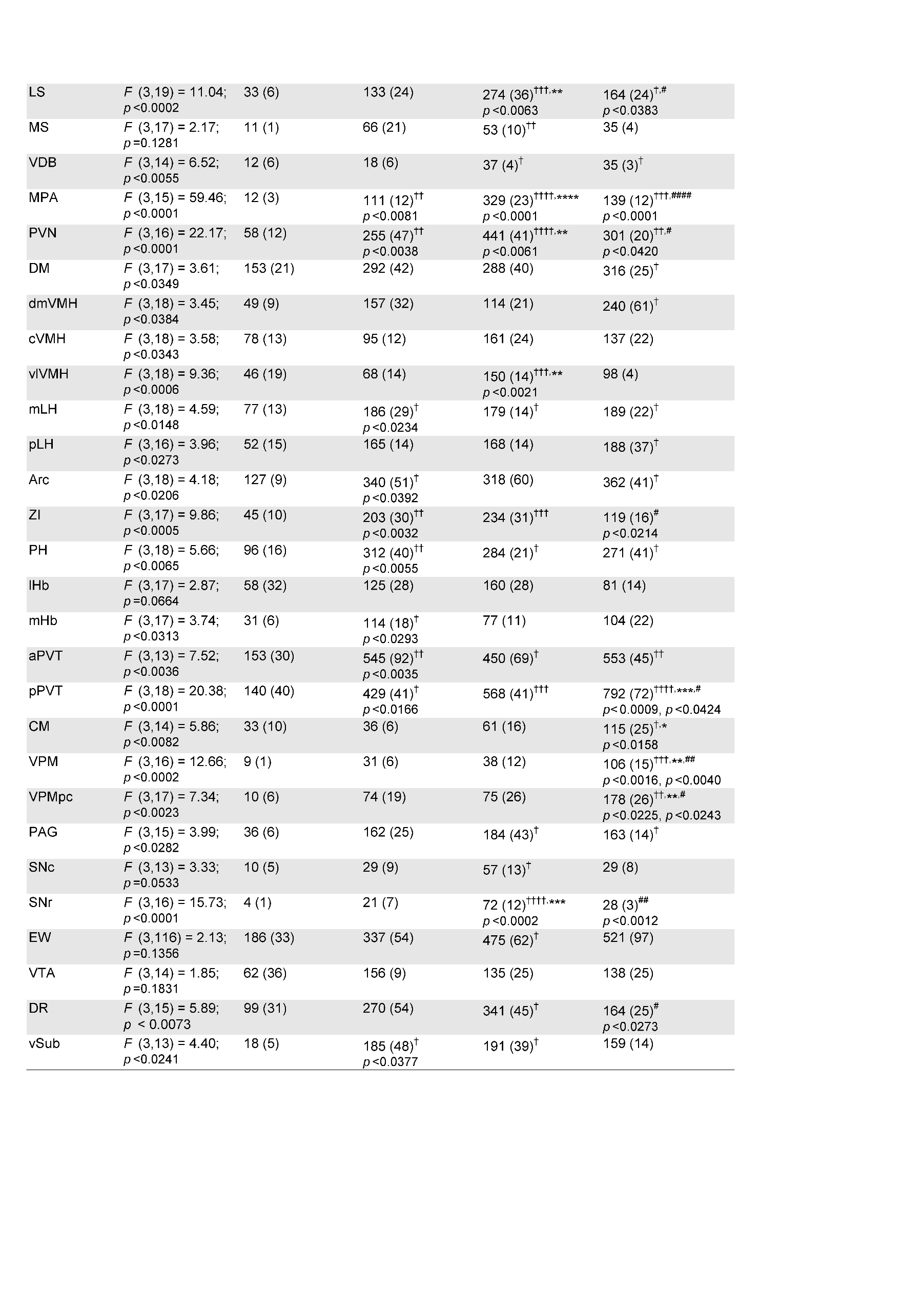


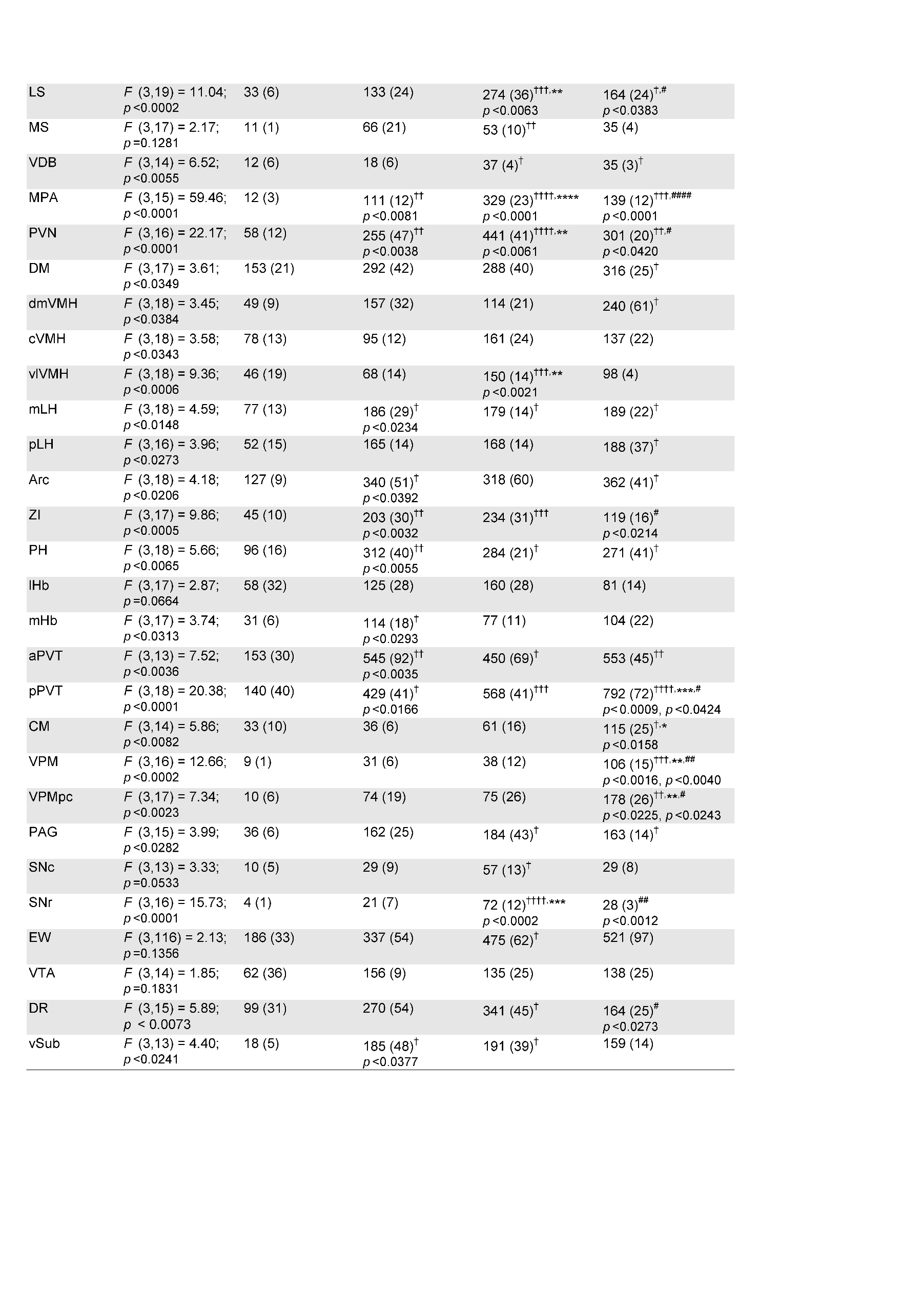
Data presented as average number of Fos-positive cells per mm^2^ ± *SEM*. Naïve *n*=3-4; Control *n*=4-7; Stress only *n*=5-6; Stress-binge *n*=5-8. ^†^*p*<0.05, ^††^*p*<0.01, ^†††^*p*<0.001, ^††††^*p*<0.0001 versus naïve group; **p* < 0.05, ***p*<0.01, ****p*<0.001, *****p*<0.0001 versus control group; ^#^*p*< 0.05 ^##^*p*<0.001 ^####^*p*<0.0001 versus stress only group (one-way ANOVA with Tukey post hoc multiple comparisons or Kruskal-Wallis followed by Dunn’s multiple comparisons for non-normally distributed datasets). Brain region abbreviations can be found on supplementary table S2 legend.

**Pattern of neuronal activation differs between stress groups**

The number of Fos-positive cells in the stress only group was higher than the control group in the lateral septum (Fig. S3A), medial preoptic area (Fig. S3B), PVN (Fig. S3C), ventrolateral VMH (Fig. S3D), and substantia nigra reticulata (SNr) (Fig. S3E). In contrast, higher Fos expression in the stress-binge group versus control group was observed in the anterior insula (Fig. S3F), agranular insular cortex (Fig.S3G), bed nucleus of the stria terminalis, lateral division (lBNST) (Fig. S2H), posterior PVT (Fig. S3I), central medial nucleus of the thalamus (Fig. S3J), ventral posteromedial thalamic nucleus (VPM) (Fig. S3K), and ventral posteromedial thalamic nucleus, parvicellular portion (VPMpc) (Fig. S3L). Regions with significantly different Fos expression in the stress-binge group versus stress only group were the central amygdala (CeA, Fig. S3M, increased), zona incerta (Fig. S3N, decreased) and dorsal raphe (Fig. S3O, decreased). The dorsal peduncular cortex (Fig. S3P) was the only brain region that exhibited significantly different Fos expression in both stress groups relative to controls.


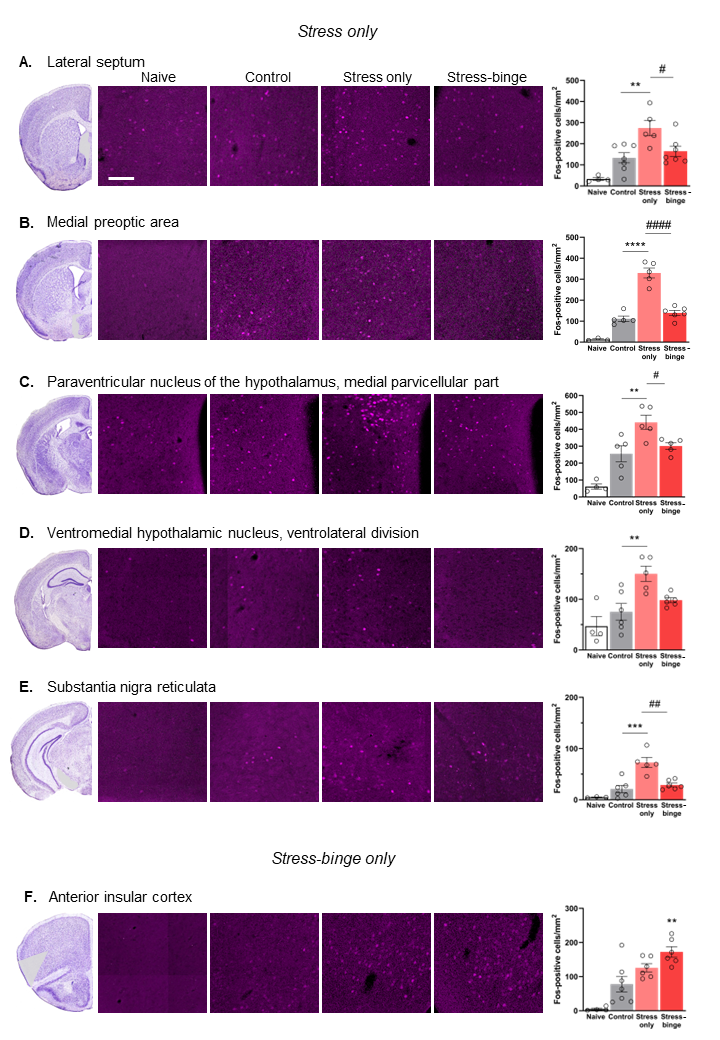


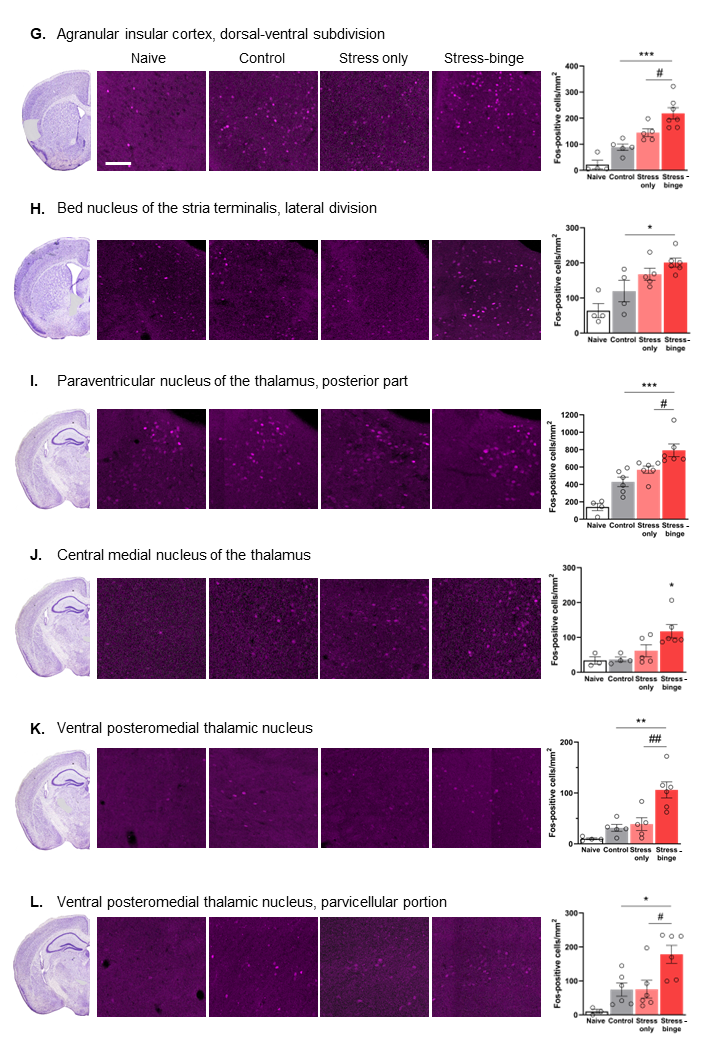

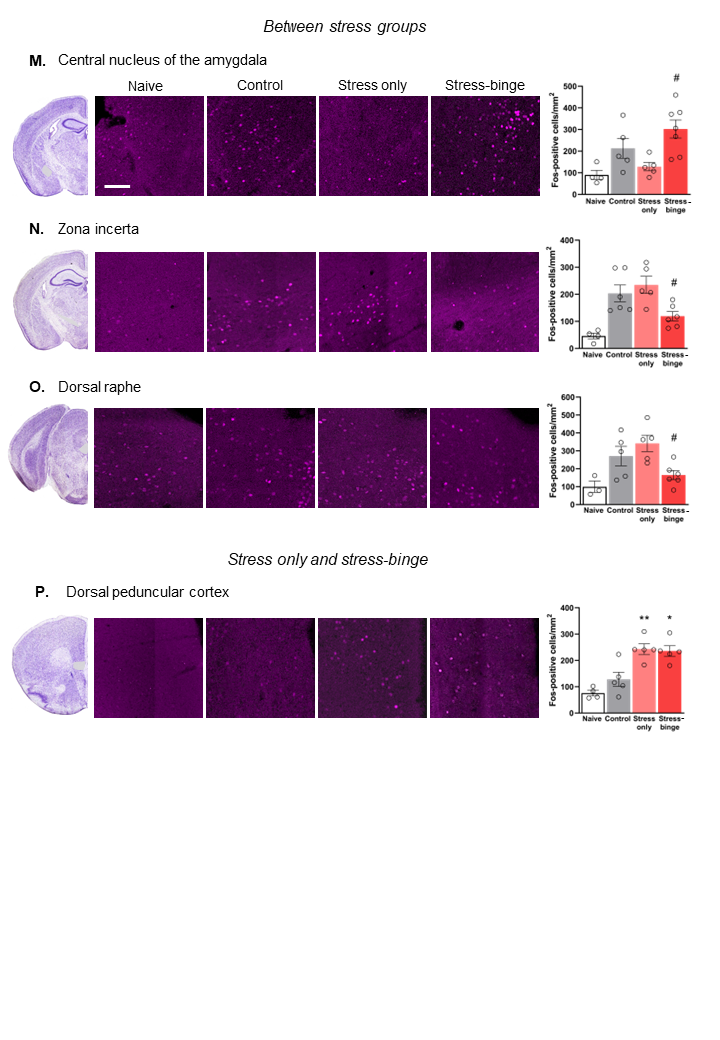


**Supplementary Figure S2.** **Distinct patterns of neuronal activation across different experimental groups.** When compared to control and stress-binge groups, brain regions from **A**-**E** showed significant increase in cellular activation following stress manipulations. **F**-**L** depicts regions that showed significant increased Fos expression following stress-binge when compared to control and stress only groups. **M**-**O** depicts regions that had distinct neuronal activation between stress only and stress-binge groups. The dorsal peduncular area (**P**) had significant increased cellular activation following both stress manipulations and stress-binge when compared to control group. Light grey delineations in the atlas images represent the approximate area of the brain region reported. Scale bar: 100μm. Data presented as mean ± SEM. One-way ANOVA followed by Tukey’s multiple comparisons test * *p* < 0.05, ** *p* < 0.01, ****p*<0.001, *****p*<0.0001 versus control group; ^#^*p*<0.05, ^##^*p*<0.01, ^####^*p*<0.0001 between stress groups. Naïve *n*=3-4, control *n*=4-7, stress only *n*=5-6, stress-binge *n=*5-8.

**Palatable food consumption is correlated with Fos expression in distinct brain regions**

Pearson correlation analysis was performed between Fos expression in distinct brain regions and highly palatable food consumption on the test day. In the control group, Fos expression in the ventral pallidum and medial septum was inversely correlated with the amount of highly palatable food consumed (ventral pallidum *r*=-0.779, *p*<0.0386, Fig. S4A-B; medial septum *r*=-0.806, *p*<0.0285, Fig. S4A, C). In the stress-binge group, a positive correlation between food consumption and the number of Fos-positive cells occurred in the medial agranular insular cortex (*r*=0.759, *p*<0.0476, Fig. S4A, D) and medial LH (*r*=0.882, *p*<0.0199, Fig. S3A, E).


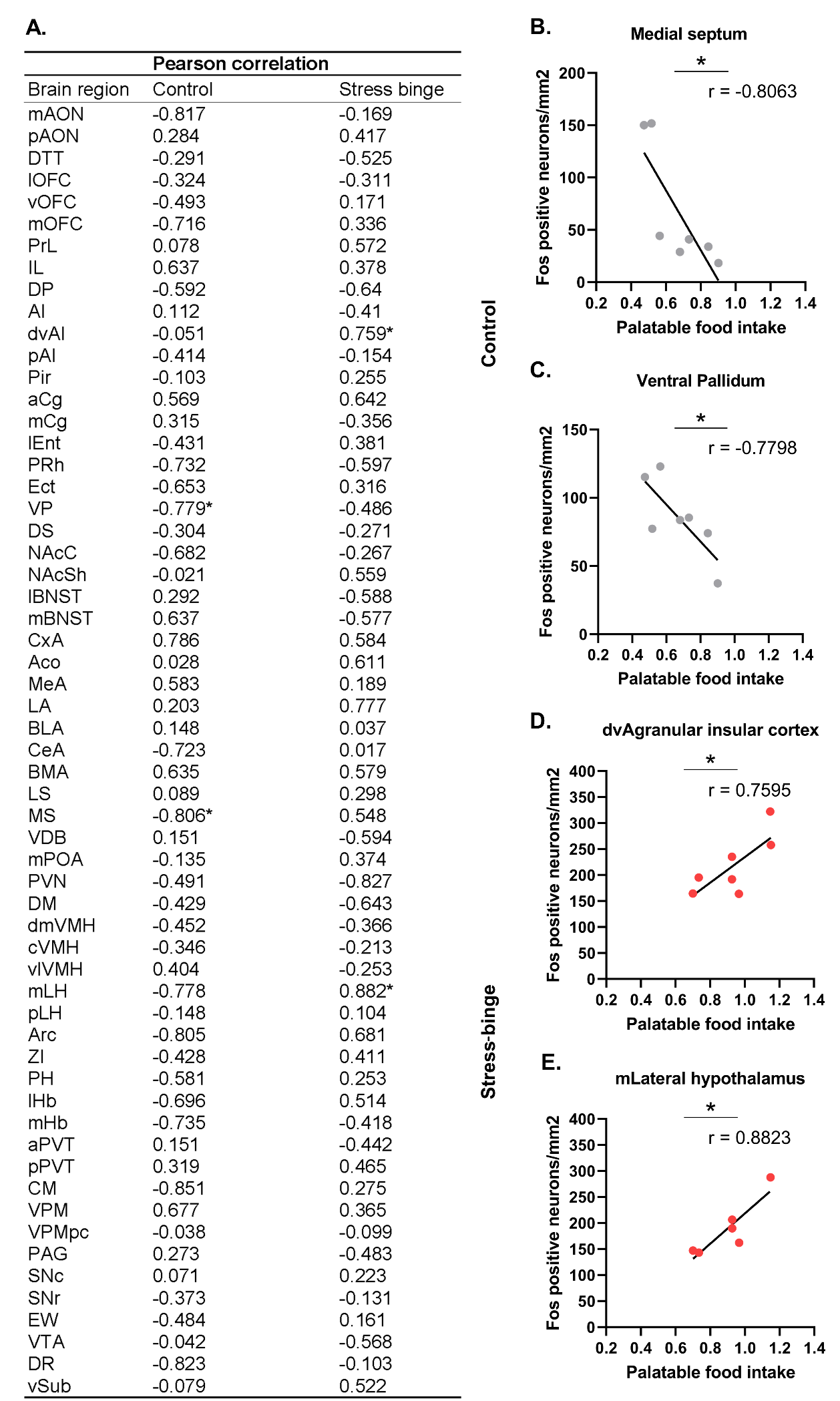


**Supplementary Figure S3.** **Correlations between Fos expression and highly palatable food consumption in control or stress-binge group**. **A**. Pearson’s correlations were performed between the amount of highly palatable food consumption and Fos expression in 59 brain regions from control and stress-binge groups. **B-C**. Inverse correlations in the ventral pallidum (*p*=0.0386) and medial septum (*p*=0.0285) from control group. **D-E**. Correlations in the medial agranular insular cortex, dorsal-ventral subdivision (*p*=0.0476), and lateral hypothalamus, medial division (*p*=0.0199) from stress-binge group. Data presented as the Pearson correlation coefficient (*r*), **p*<0.05. *n*=6-8 per group. aCg, anterior cingulate cortex; ACo, anterior cortical amygdaloid nucleus; AI, anterior insula; aPVT, paraventricular nucleus of the thalamus, anterior part; Arc, arcuate hypothalamic nucleus; BLA, basolateral amygdala; BMA, basomedial amygdala; CeA, central nucleus of the amygdala; CM, central medial nucleus of the thalamus; cVMH, ventromedial hypothalamic nucleus, central division; CxA, cortex-amygdala transition zone; DM, dorsomedial hypothalamic nucleus; dmVMH, ventromedial hypothalamic nucleus, dorsomedial division; DP, dorsal peduncular cortex; DR, dorsal raphe; DS, dorsal striatum; DTT, dorsal tenia tecta; dvAI, agranular insular cortex, dorsal-ventral subdivision; Ect, ectorhinal cortex; EW, Edinger-Westphal; IL, infralimbic cortex; LA, lateral amygdala; lBNST, bed nucleus of stria terminalis, lateral division; lEnt, lateral entorhinal cortex; lHb, lateral habenula; lOFC, orbitofrontal cortex, lateral subdivision; LS, lateral septum; MS, medial septum; mAON, anterior olfactory nucleus, medial part; mBNST, bed nucleus of the stria terminalis, medial division; mCg, cingulate cortex, medial part; MeA, medial nucleus of the amygdala; mHb, medial habenula; mLH, lateral hypothalamus, medial division; mOFC, orbitofrontal cortex, medial subdivision; mPOA, medial preoptic area; NAcC, nucleus accumbens core; NAcSh, nucleus accumbens shell; PAG, periaqueductal gray; pAI, agranular insular cortex, posterior part; pAON, anterior olfactory nucleus, posterior part; PH, posterior hypothalamic area; Pir, piriform cortex; pLH, lateral hypothalamus, posterior division; pPVT, paraventricular thalamus, posterior part; PRh, perirhinal cortex; PrL, prelimbic cortex; PVN, paraventricular nucleus of the hypothalamus, medial parvicellular part; SNc, substantia nigra compacta; SNr, substantia nigra reticulata; VDB, nucleus of the vertical limb of the diagonal band; vlVMH, ventromedial hypothalamic nucleus, ventrolateral division; vOFC, orbitofrontal cortex, ventral subdivision; VP, ventral pallidum; VPM, ventral posteromedial thalamic nucleus; VPMpc, ventral posteromedial thalamic nucleus, parvicellular portion; vSub, ventral subiculum; VTA, ventral tegmental area; ZI, zona incerta.

**References**

1. Paxinos G, Franklin KBJ. The mouse brain in stereotaxic coordinates. 3^rd^ ed. Academic Press: Sydney, NSW. 2008.

2. Walker LC, Kastman HE, Krstew EV, Gundlach AL, Lawrence AJ. Central amygdala relaxin-3/relaxin family peptide receptor 3 signalling modulates alcohol seeking in rats. Br J Pharmacol. 2017;174: 3359-69.

3. Campbell EJ, Maddern XJ, Lawrence AJ. Repeated, moderate footshock reduces the propensity to relapse to alcohol seeking in female, but not male, iP rats. Behav Neurosci. 2021;135: 771-81.

4. Kimborough A, Lurie DJ, Collazo A, Kreifeldt M, Sidhu H, Macedo GC, et al. Brain-wide functional architecture remodelling by alcohol dependence and abstinence. Proc Natl Sci USA*.* 2020;117:2149-59.

5. Wang F, Flanagan J, Su N, Wang LC, Bui S, et al. RNAscope: A novel in situ RNA analysis platform for formalin-fixed, paraffin-embedded tissues. J Mol Diagn*.* 2012;14: 22-29.

6. Walker LC, Berizzi AE, Chen NA, Rueda P, Perreau VM, Huckstep, et al. Acetylcholine muscarinic M4 receptors as a therapeutic target for alcohol use disorder: Converging evidence from humans and rodents. Biol Psych. 2020*;*88: 898-909.

7. Walker LC, Hand LJ, Letherby B, Huckstep KL, Campbell EJ, Lawrence AJ. Cocaine and amphetamine regulated transcript (CART) signalling in the central nucleus of the amygdala modulates stress-induced alcohol seeking. Neuropsychopharmacol. 2021;46: 325-33.

8. Beier KT, Steinberg EE, DeLoach KE, Xie S, Miyamichi K, Schwarz L, et al. Circuit Architecture of VTA Dopamine Neurons Revealed by Systematic Input-Output Mapping. Cell 2015;162: 622-34.

9. Saunders A, Johnson CA, Sabatini BL. Novel recombinant adeno-associated viruses for Cre activated and inactivated transgene expression in neurons. Front Neural Circuits. 2012;6: 47.
